# Using Expanded Natural Killer Cells as Therapy for Invasive Aspergillosis

**DOI:** 10.3390/jof6040231

**Published:** 2020-10-17

**Authors:** Win Mar Soe, Joan Hui Juan Lim, David L. Williams, Jessamine Geraldine Goh, Zhaohong Tan, Qi Hui Sam, Sanjay H. Chotirmall, Nur A’tikah Binte Mohamed Ali, Soo Chin Lee, Ju Ee Seet, Sharada Ravikumar, Louis Yi Ann Chai

**Affiliations:** 1 Division of Infectious Diseases, University Medicine Cluster, National University Health System, Singapore 119228, Singapore; winmarsoe18@gmail.com (W.M.S.); valdemar25@hotmail.com (J.H.J.L.); jessamine_goh@sris.a-star.edu.sg (J.G.G.); Zhaohong_tan@nuhs.edu.sg (Z.T.); sharada_ravikumar@nuhs.edu.sg (S.R.); 2 Department of Haematology-Oncology, National University Cancer Institute, Singapore, National University Health System, Singapore 119074, Singapore; mdcleesc@nus.edu.sg; 3 Department of Surgery and Center for Inflammation, Infectious Disease and Immunity, James H. Quillen College of Medicine, East Tennessee State University, Johnson City, TN 37614, USA; williamd@mail.etsu.edu; 4 Department of Biochemistry, National University of Singapore, Singapore117596, Singapore; qihui.sam@nus.edu.sg; 5 Lee Kong Chian School of Medicine, Nanyang Technological University, Singapore 639798, Singapore; schotirmall@ntu.edu.sg (S.H.C.); atikah@ntu.edu.sg (N.A.B.M.A.); 6 Department of Pathology, National University of Singapore, Singapore 117597, Singapore; ju_ee_seet@nuhs.edu.sg; 7 Department of Medicine, Yong Loo Lin School of Medicine, National University of Singapore, Singapore 119228, Singapore

**Keywords:** *Aspergillus*, antifungal immunity, fungal infection, immune evasion, immune recognition, expanded natural killer cells

## Abstract

Invasive aspergillosis (IA) is a major opportunistic fungal infection in patients with haematological malignancies. Morbidity and mortality rates are high despite anti-fungal treatment, as the compromised status of immune system prevents the host from responding optimally to conventional therapy. This raises the consideration for immunotherapy as an adjunctive treatment. In this study, we evaluated the utility of expanded human NK cells as treatment against *Aspergillus fumigatus* infection in vitro and in vivo. The NK cells were expanded and activated by K562 cells genetically modified to express 4-1BB ligand and membrane-bound interleukin-15 (K562-41BBL-mbIL-15) as feeders. The efficacy of these cells was investigated in *A. fumigatus* killing assays in vitro and as adoptive cellular therapy in vivo. The expanded NK cells possessed potent killing activity at low effector-to-target ratio of 2:1. Fungicidal activity was morphotypal-dependent and most efficacious against *A. fumigatus* conidia. Fungicidal activity was mediated by dectin-1 receptors on the expanded NK cells leading to augmented release of perforin, resulting in enhanced direct cytolysis. In an immunocompromised mice pulmonary aspergillosis model, we showed that NK cell treatment significantly reduced fungal burden, hence demonstrating the translational potential of expanded NK cells as adjunctive therapy against IA in immunocompromised patients.

## 1. Introduction

Invasive aspergillosis (IA) results in significant mortality and morbidity in immunocompromised hosts, in particular patients with haematological malignancies receiving chemotherapy or conditioning regimens for stem cell transplantation. Despite the availability of newer anti-fungal agents and improved strategies of antifungal prophylaxis, IA mortality rates can be in excess of 35% [1]. 

To date, there is increased understanding of the roles played by the host innate and adaptive immune responses against *Aspergillus*, as well as the defense mechanisms which are iatrogenically subverted during chemotherapy and immunosuppressive treatment [2,3]. The limited response to treatment may be attributable to the inability of host immunity to respond appropriately to the pathogens. In response to this, efforts have been invested in recent years on innovative approaches to immunotherapy and augmentation of the host immunity. 

Natural killer (NK) cells are known to play a central effector role against viral pathogens. Recent research has described the involvement of NK cells in the mounting of host response against fungi [4,5,6,7]. This raises the prospect that NK cell therapy might potentially be efficacious as an immunotherapeutic option against *A. fumigatus,* especially in patients with haematologic malignancies in lieu of their cytotoxic efficacy against both tumour- and pathogen-infected cells [8,9,10]. Despite the purported efficacy of NK cells, their relative paucity, short life span, and the need for multiple signals for sustained proliferation, activation, and survival pose as challenges associated with using NK cells for immunotherapeutic purposes [11]. A recent development pioneered the expansion of NK cells in the presence of myeloid cells which had been genetically modified to express membrane bound IL15 and 4-1BB ligand (CD137L). These expanded NK cells are highly active, loaded with cytotoxic granules, perforin, and granzymes standing ready for cytotoxic activity [12]. Here, we investigate the antifungal activity of such expanded and activated natural killer cells against different morphotypes of *A. fumigatus* both in vitro and in vivo. Our findings demonstrate that adoptive transfer of expanded NK cells is a viable and novel treatment modality against *Aspergillus* infection.

## 2. Materials and Methods

### 2.1. Expansion of Natural Killer Cells

Activated NK cells expansion is as described in previous studies [13]. In short, peripheral blood mononuclear cells (PBMC) were incubated with 100 Gy-irradiated K562-mbIL15-41BBL cells in Stem Cell Growth Medium (SCGM; CellGenix, Freiburg, Germany) supplemented with 10% FBS and 10 U/mL rIL-2. Depletion of CD3 positive T cells was performed after 1 week using CD3 DynaBeads (Invitrogen, Carlsbad, CA, USA). Purified NK cells were expanded further with rIL-2 supplemented SCGM for 1 more week. After the expansion, purity of expanded natural killer cells was assessed by flow cytometry. The cells were analyzed using FACS Calibur flow cytometer (BD Biosciences, San Jose, CA, USA). The purity of the expanded NK cells was confirmed using CD3-APC (Miltenyi, Singapore) and CD56-FITC (Miltenyi, Singapore) and the cell viability was more than 92%. The expanded NK cells used in this study contained less than 5% of CD3^+^CD56^−^ T cells. The PBMC and the expanded NK cells derived from them were a kind gift from Dr. Dario Campana’s laboratory at Department of Pediatrics, Yong Loo Lin School of Medicine, National University of Singapore. 

### 2.2. Preparation of Aspergillus fumigatus

Preparation of *A. fumigatus* was done using a previously well characterized *A. fumigatus* clinical strain (V05-27) [14]. The *Aspergillus* was grown on chloramphenicol supplemented Sabouraud glucose agar slants for 4–7 days at 37 °C. *A. fumigatus* spores were harvested by scraping the surface of slants and re-suspending in 0.05% Tween 20 in PBS. Unwanted hyphae were removed by passing the suspension through sterile gauze. The conidial suspension was washed twice and re-suspended in RPMI1640. 

### 2.3. Pulmonary Aspergillosis Mice Model

All the animals were housed in the animal facility at Biological Resource Centre, Singapore. Animals were handled following Singapore’s “Guidelines on the Care and Use of Laboratory Animals for Scientific Purposes”. Experiments were conducted after approval by Institutional Animal Care and Use Committee (IACUC) under protocol 181308. 

### 2.4. Immunosuppression

**Figure 1:**
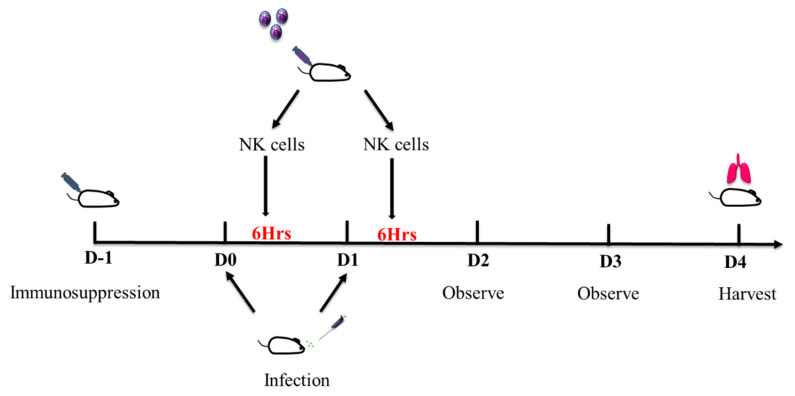
Pulmonary Aspergillosis mice model. Schematic diagram of administration of expanded natural killer cells and *A. fumigatus*.

Eight-week-old wild type male Balb/c mice were obtained from InVivos. Mice were immunosuppressed with subcutaneous injection of cortisone acetate (250 mg/kg/200 µL) and intra-peritoneal injection of cyclophosphamide (250 mg/kg/100 µL) on day -1. On D0, 20 µL of *A. fumigatus* spores (1 × 10^6^ fungal cells) were instilled into the nose of the mice after they were lightly anesthetized with isoflurane. Six hours after the infection, 200 µL of expanded NK cells (1 × 10^7^ CFU/mL) suspended in PBS were injected into tail veins of the treated group, while 200 µL of PBS was injected into tail veins of the control group. The infection and treatment procedures were repeated on D1. Mice were observed on D2 and D3. On D4, mice were sacrificed, and lungs were harvested for fungal load quantification. Augmentin 0.25 mg/mL was given in drinking water throughout the duration of the experiment to decrease the risk of superimposed bacterial infection due to immunosuppression [15]. The timeline of immunosuppression and infection is as depicted in Figure 1. 

### 2.5. Fungal Load Quantification 

Lung tissues were removed from each mouse, homogenized and diluted in serial 10-fold dilutions. These were plated on Sabouraud agar plates supplemented with chloramphenicol and incubated at 35 °C. After 3 days, fungal colony forming units (CFUs) were enumerated. 

### 2.6. Histology

Lung tissues were removed from each mouse and placed in 10% formalin. Lung sections were stained with Hematoxylin and eosin (HE) and Gomori methenamine silver (GMS) stains to identify fungal structures and visualize host response. Slides were viewed on an Olympus BX41 microscope and representative images captured at ×200 original magnification using an Olympus DP22 digital camera attached to the microscope. 

### 2.7. Fungal Killing Assay

Fungal killing activity of the expanded NK cells was assessed by using a colorimetric assay with (2,3-bis-[2-methoxy-4-nitro-sulfophenyl]-2*H*-tetrazolium-5-carboxyanilide) sodium salt (XTT; Sigma Aldrich, St Louis, MO, USA). *A. fumigatus* spores were plated on 48-well plates at a concentration of 2.5 × 10^6^ CFU/mL per well. The spores were incubated for 8–10 h to obtain hyphae and 4 h for swollen conidia. Expanded NK cells were seeded into wells containing fungi at a concentration of 5 × 10^6^ cells/mL with effector: target ratio of 2:1. Wells with hyphae or swollen conidia of *A. fumigatus* alone were used as positive control while wells with expanded NK cells only were used as negative controls. Expanded NK cells were co-incubated with *A. fumigatus* for 6 h; supernatant was collected for cytokine measurement. XTT assay was performed by washing the cells with sterile cold water to lyse NK cells. XTT was added at a concentration of 0.25 mg/mL and incubated for 3 more hours at 37 °C with 5% CO_2_. 100 µL of the solution was transferred to a 96-well plate and the absorbance was read at a wavelength of 450 nm with 690 nm reference. Percent fungal damage was calculated by using the formula: $\left(1-\frac{x}{c}\right)\times 100$, where x is the absorbance of experimental wells and c is the absorbance of positive control wells.

### 2.8. Dectin-1 Inhibition Assay

NK cells were incubated with the dectin-1 receptor antagonist laminarin (250 µg/mL) for 1 h before being seeded into the well containing the *A. fumigatus*. Laminarin was kindly provided by Dr David Williams (East Tennessee State University, Johnson City, TN, USA). The XTT assay was then carried out as described above.

### 2.9. Cytokine and Perforin Quantification 

Perforin, granzyme B, IFN-γ, TNF-α, IL1β and IL6 release in the supernatants were measured using commercially available enzyme-linked immunosorbent assay (ELISA kits; Perforin—Abcam, Cambridge, MA, USA; Granzyme B—R&D Systems, Minneapolis, MN, USA; IFN-γ, IL1β, IL6, TNF-α—eBioscience, San Diego, CA, USA) according to manufacturers’ instructions. The lowest detection limits were as follow: perforin, 40 pg/mL; granzyme B, 39 pg/mL; IFN-γ 4 pg/mL; TNF-α 4 pg/mL; IL1β 4 pg/mL; IL6, 2 pg/mL. 

### 2.10. Statistics 

In vitro experiments were performed in duplicates. Results were pooled from at least five sets of separate experiments. In vivo experiments were performed using at least five mice in each group. Results were pooled from three sets of mice experiments. The results were analyzed using GraphPad Prism (Version 7, San Diego, CA, USA) and the Wilcoxon signed rank test was used. The level of significance was set at *p* < 0.05.

## 3. Results

### 3.1. Expanded Natural Killer Cells had Enhanced Fungicidal Activity Against Aspergillus conidia

**Figure 2:**
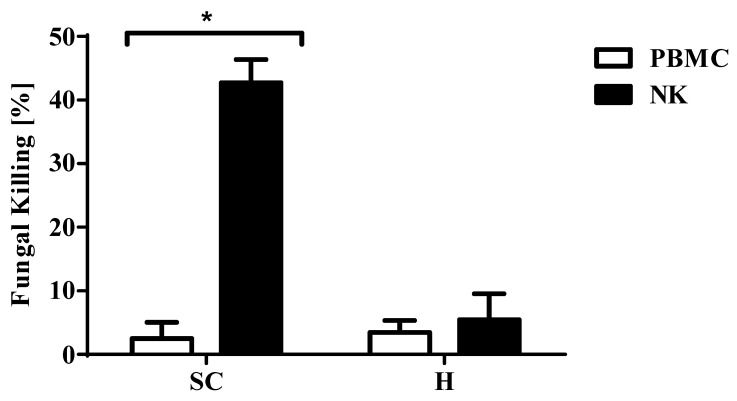
Killing activity of expanded NK cells as determined by XTT assay. The killing effect of expanded NK cells and PBMC on the two morphotypes of *A. fumigatus*, namely, SC—swollen conidia and H—hyphae was determined. The Effector: Target (NK cell: *Aspergillus* or PBMC: *Aspergillus*) ratio used was 2:1. Data represent the mean of duplicate measurements (± SEM) pooled from 5 experiments. * *p* < 0.05 compared to PBMC.

To determine the antifungal activity of the expanded NK cells against two morphotypes of *A. fumigatus*, we performed XTT assay by co-incubating expanded NK cells with the fungi at a low effector to target (E:T) ratio of 2:1 for 6 h. This was compared against the fungicidal activity of PBMC at the corresponding E: T ratio (Figure 2). Expanded NK cells had markedly increased fungal killing activity of 42% (±3%) on swollen conidia over that of PBMC’s 2%. Results were pooled from at least five experiments with *p* < 0.05. There was no difference in *A. fumigatus* hyphae fungicidal activity between expanded NK cells (4.3%) and PBMC (3.4%). 

### 3.2. The Antifungal Activity of Expanded NK Cells Is Not Attributable to Enhanced Proinflammatory Cytokine Response

**Figure 3:**
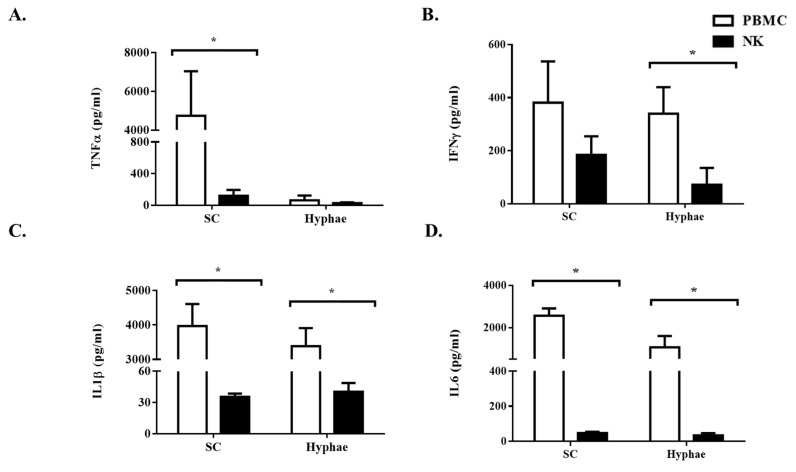
Cytokine production by expanded NK cells. (**A**)—TNF-α; (**B**)—IFN-γ; (**C**)—IL1β; (**D**)—IL6 in the supernatants of expanded NK cells and PBMC exposed to different morphotypes of *A. fumigatus* were assessed by ELISA. Expanded NK cells and PBMC were co-incubated with live *Aspergillus* morphotypes for 6 h. Data shown is representative of the mean of duplicate readings (± SEM) pooled from 5 experiments. * *p* < 0.05 for PBMC compared to expanded NK cells.

We queried if the antifungal activity of the expanded NK cells might be mediated by proinflammatory cytokines. Tumor necrosis factor-alpha (TNF-α) and interferon-gamma (IFN-γ) are known to play central roles in effecting the host effector response against *Aspergillus* [5,16]. Expanded NK cells did not produce TNF-α against the various *Aspergillus* morphotypes as compared to PBMC (Figure 3A). Interferon-gamma production by the expanded NK cells was not higher than PBMC (Figure 3B) and were in fact suppressed against swollen conidia and hyphae. Similarly, IL1β and IL6 levels were detected at modest levels in NK cells incubated with *Aspergillus*, in contrast to PBMC (Figure 3C,D). The findings were indicative that the observed enhanced antifungal efficacy of the NK cells was independent to that of an enhanced cytokine response. 

### 3.3. Expanded NK Cells Release High Level of Perforin 

**Figure 4:**
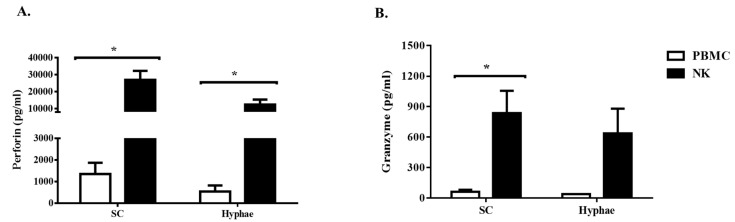
Production of cytotoxic granules by expanded NK cells. (**A**)—Perforin, (**B**)—Granzyme B in the supernatants of expanded NK cells and PBMC exposed to swollen conidia (SC) and hyphae (H) of *A. fumigatus* were assessed by ELISA. Data represent the mean of duplicate measurements (± SEM) pooled from 3 experiments. * *p* < 0.05 compared to PBMC.

Another important modality for cytotoxic capacity of NK cells is through the release of cytotoxic granules, perforin, which induces pores in the target cell membrane, and granzymes that enter target cells and induce death by activating caspase [17,18]. The expanded NK cells released significantly high levels of perforin when they were exposed to swollen conidia (Figure 4A). However, perforin release was found to be suppressed in NK cells co-incubated with the *Aspergillus* hyphae. While granzyme B release was significantly higher in NK cells in comparison with PBMC, the levels were found to be much lower when compared with the release of perforin (Figure 4B). In our study, fungal killing capacity of NK cells on swollen conidia was found to be independent of granzyme B, which highlights the functional importance of perforin as an effector cytotoxic molecule against *Aspergillus*. 

### 3.4. Effect of Dectin-1 Inhibition on Expanded NK Cells 

**Figure 5:**
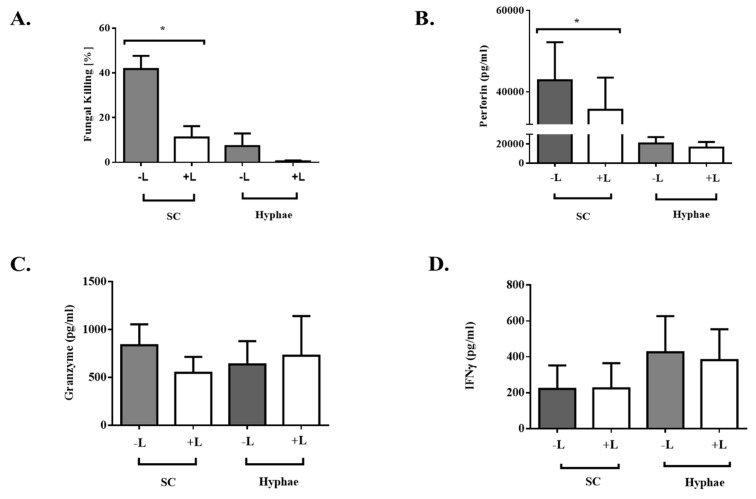
Dectin-1 inhibition assay with laminarin. Expanded NK cells were treated with laminarin for 1 h before exposure to the *Aspergillus* morphotypes (swollen conidia—SC and Hyphae—H) for 6 h. (**A**) XTT assay showing killing of swollen conidia and hyphae by cells treated with laminarin compared to the untreated cells (* *p* < 0.05). (**B**) Perforin levels in cells treated with laminarin compared to untreated cells (* *p* < 0.05). (**C**) Granzyme B levels and (**D**) IFN-γ production following blockade of dectin-1 receptors with laminarin. Data shown is representative of the mean of duplicate readings (± SEM) pooled from 3 experiments.

Dectin-1 is a well-described C type lectin receptor for beta-glucan in the fungal cell wall. However, the role of dectin-1 on NK cells is not well studied and we queried if dectin-1 might be involved in mediating the cytolytic activity of the expanded NK cells. Blocking dectin-1 with laminarin reduced the fungal killing capacity of expanded NK cells, and this was most clearly seen with the swollen conidia (Figure 5A). Correspondingly, there was a significant reduction in perforin levels from laminarin-treated NK cells with swollen conidia (Figure 5B). On the other hand, granzyme B levels were found to be minimally affected by presence of laminarin (Figure 5C). The production of IFN-γ was independent of dectin-1 blockade (Figure 5D), which was in line with the above findings that IFN-γ and other proinflammatory cytokines were not the mediators of the fungicidal activity of expanded NK cells.

These results point to the involvement of the dectin-1 pathway in mediating the enhanced cytolytic activity of expanded NK cells through release of perforin against swollen *Aspergillus* conidia. 

### 3.5. In vivo Antifungal Activity of Expanded NK Cells Against Invasive Pulmonary Aspergillosis 

**Figure 6:**
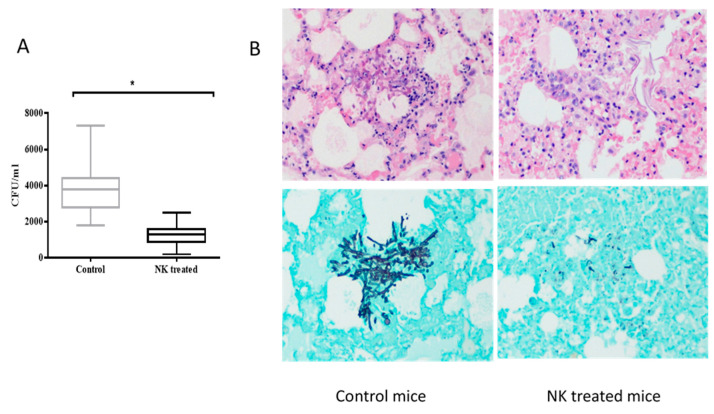
Fungal burden in neutropenic mice treated with expanded NK cells. Mice were infected with *Aspergillus* followed by treatment with either expanded NK cells or PBS for 2 consecutive days. The lungs were harvested following the infection and treatment. (**A**) The fungal burden in mice treated with expanded NK cells compared to the PBS treated controls. 20 mice each were pooled from 3 experiments + SEM (* *p* < 0.05). (**B**) Lungs from 5 mice each in NK or PBS treated groups were stained with H&E (top panel) and GMS (bottom panel) and ×200 magnification was used to identify fungal structures and visualize host response.

To validate the efficacy of expanded NK cells against invasive pulmonary aspergillosis in vivo, immunocompromised neutropenic mice were infected with *A. fumigatus*, and treated with expanded NK cells. Mice treated with the expanded NK cells had significantly lower fungal burden (40% reduction) in the lungs when compared to untreated mice (Figure 6). Histologically there were less fungi and inflammatory changes in the lungs of NK cells-treated mice as compared to the untreated. 

## 4. Discussion

We have demonstrated the utility of expanded and activated NK cells in the treatment of invasive aspergillosis in vivo. Natural killer cells play a pivotal role in our host defense system against major pathogens and is a candidate as an immunotherapeutic agent. Earlier studies had utilized primary NK cells activated by IL-2 and had mostly reported only in vitro cytotoxicity against the fungi [9]. However, the purported utility of NK cells had been limited by the challenges in obtaining sufficient numbers of pure NK cells suitable for manipulation and expansion [19].

The NK cells employed here had been expanded in the presence of myeloid cells that were genetically modified to express membrane bound IL-15 and 4-1BB ligand (CD137L). Expansion of NK cells through this method results in a 1000-fold enhancement in the yield of CD56 + CD3- cells [13,20]. These expanded NK cells maintain high-level activation while still possessing properties of immunophenotypic diversity and specific natural cytotoxicity of peripheral blood NK cells. It was shown that more than 80% of these highly activated expanded NK cells have high expression of perforin, granzyme A, and granzyme B molecules as well as NKG2D and other natural cytotoxicity receptors such as NKp30 which was linked to direct recognition and killing of fungal cells [21,22]. To demonstrate the potency of the cells derived through this method, we had first attempted effector to target (E:T) ratio of 5:1 which showed clear anti-fungal activity, and next using E:T ratio of just 2:1 sufficed in effecting fungicidal activity against *A. fumigatus*, and over that of the peripheral blood mononuclear cells. The superiority of this fungicidal efficacy far exceeded that of earlier studies using primary NK cells which had required E:T ratios ranging from 50:1 to 10:1 [9]. 

Conventionally, IFN-γ is pivotal in coordinating anti-viral effector response and in the context of *Aspergillus*. Interferon-gamma released by NK cells has been described as being capable of exerting direct damage to the fungi [4] and in earlier studies using mice-derived NK cells, IFN-γ was seen to play a prominent role in mediating anti-fungal activity [23]. In our study here, using expanded human NK cells alternatively activated through K562-41BBL-mbIL-15, a primary modality of cytotoxicity against *Aspergillus* was through the release of perforin. Interferon-gamma production, while still evident, was not augmented in the presence of the activated NK cells; nonetheless, we cannot totally disregard its possible role. The levels of IFN-γ produced may be sufficient to control the earlier phase of swollen conidia, but not the later phase where hyphae are more predominant. Correspondingly, the high levels of IFN-γ produced by the control PBMC could be explained by the presence of monocytes and lymphocytes in addition to the NK cells. 

The other recognized effector mechanism of NK cells would have been through the degranulation of cytotoxic proteins as represented by perforin [24]. Our findings highlight that expanded NK cells had the capacity for augmented perforin release with increased cytolytic activity against *A. fumigatus.* The activity of the expanded NK cells was morphotype-dependent: they were most active against swollen *A. fumigatus* conidia over the hyphae. We had also observed a tendency for an immunosuppressive effect exerted by the *A. fumigatus* hyphae, resulting in downregulation of IFN-γ [5,9]. This capacity of the *Aspergillus* hyphae to suppress effector cytokines may surmount to subversion of the host antifungal defense [25,26], further highlighting the importance for cytokine-independent host defence strategies such as cytotoxic degranulation. Having said that, our finding is in contrast to that of Schmidt et al. [9], which showed activity between NK cells and *Aspergillus* hyphae, but not against resting or germinating conidia. We can only postulate that the differential activity against the fungal morphotypes lies in the different modalities of NK cell activation and end-product-primary and IL2-stimulated NK cells versus expansion and activation through K562-41BBL-mbIL-15; as further supported by the very different E: T efficacy of the NK cells produced by the two groups. The observed limited activity of the NK cells against the hyphal form here reinforces the needful administration of concurrent anti-fungal therapeutics ideally effective against swollen conidia and hyphae to optimize treatment outcomes [27]. 

As beta-glucan is exposed on surfaces of swollen *Aspergillus* conidia, we had surmised that the beta-glucan receptor dectin-1 might be involved [28]. NK cells originating from mice were reported to have minimal, if any, expression of the dectin-1 receptor [29], though NK cells had been described to be able to respond to beta-glucan mediated through dectin-1 expressed on antigen-presenting cells [30]. Eliciting a role for the dectin-1 receptor here, its blockade by laminarin led to reduction in perforin release and resultant loss of cytotoxicity against swollen *Aspergillus* conidia over hyphae. This morphotypal-dependent anti-fungal activity is well accounted for by the observation that beta-glucan, the ligand for dectin-1, is most profoundly expressed on swollen conidia during germ tube formation and decreased with extended hyphal growth [31]. Having said that, the swollen *Aspergillus* conidia also exposes a polysaccharide exoskeleton including alpha-glucan, chitin, and galactomannan, which invokes a robust inflammatory response and may well be involved in mediating recognition and killing by the activated NK cells, besides beta-glucan elicited here [32,33,34]. In support of this observation, dectin-1 had been reported to mediate the induction of perforin in dendritic cells [35], though in the context of fungi, only two NK activating receptors NKp30 and NKp46 have been implicated in granule-dependent activity [24]. To this knowledge, we now attribute an additional novel role to the beta-glucan receptor, dectin-1, expressed on expanded NK cells in mediating anti-fungal cytotoxicity through activation of degranulation by perforin. 

Patients undergoing allogenic stem cell transplantation and induction chemotherapy for acute leukemia have the highest risks for invasive aspergillosis during the periods of iatrogenic immune nadir period [36]. In this setting of a perfect storm, invasive fungal infection opportunistically declares in the midst of profound immunosuppression whereby leucocytes and NK cell numbers are close to negligible, the capacity of the host to mount effective anti-fungal countermeasures is severely compromised even in the presence of established antifungals [3]. Under these circumstances we have shown, both in vitro and in vivo, that administration of adoptive immunotherapeutic transfer of expanded and activated NK cells is efficacious against invasive aspergillosis. Nonetheless, we are also mindful to highlight that this novel modality of treatment ought to complement anti-fungal drug treatment administered concurrently at the bedside. The expanded NK cells are already being used as treatment against leukemia and Ewing’s sarcoma in lieu of its impressive cytolytic profile and is currently being further studied in FDA-approved clinical trials [37]. This important finding has a potential to be translated as an adjunctive immunotherapeutic against invasive aspergillosis in select patient cohorts.
